# Suspected stenosis in Caroli syndrome: when cholangioscopy can be the game changer

**DOI:** 10.1016/j.vgie.2024.03.004

**Published:** 2024-03-19

**Authors:** Dario Gambaccini, Giusi Desirè Sciumè, Luigi Ruggiero, Veronica Natali, Gherardo Tapete, Valeria Bolognesi, Emanuele Marciano

**Affiliations:** 1 Endoscopy Unit, University of Pisa, Pisa, Italy; 2 Gastrointestinal Unit, Department of Medicine, Surgery and Dentistry “Scuola Medica Salernitana”, Baronissi (SA), Italy

## Abstract

Video 1 Cholangioscopy performed during ERCP.

Cholangioscopy performed during ERCP.

Caroli syndrome is a rare inherited disorder in which the patient develops segmental dilatation of the intrahepatic bile duct. The consequent stasis eases the development of stones, recurring cholangitis, risk of abscess formation, and cholangiocarcinoma.^1^^,^^2^

This is a rare case of Caroli syndrome in a 21-year-old patient who had received kidney transplant for polycystic kidney disease. He was under evaluation for liver transplant because of the extension of liver fibrosis (Metavir 4) with portal hypertension, esophageal varices, and splenomegaly.

The patient was admitted to the hospital for a suspected cholangitis. He presented with fever, abdominal pain, elevation of bilirubin, C-reactive protein, and aspartate aminotransferase. Other infectious causes were excluded. MRCP showed a stenosis of the common bile duct (Fig. 1). Therefore, the patient underwent ERCP to ascertain the nature of the stenosis, rule out the presence of neoplasia, and eventually clear the bile duct or place a stent.

**Figure 1:**
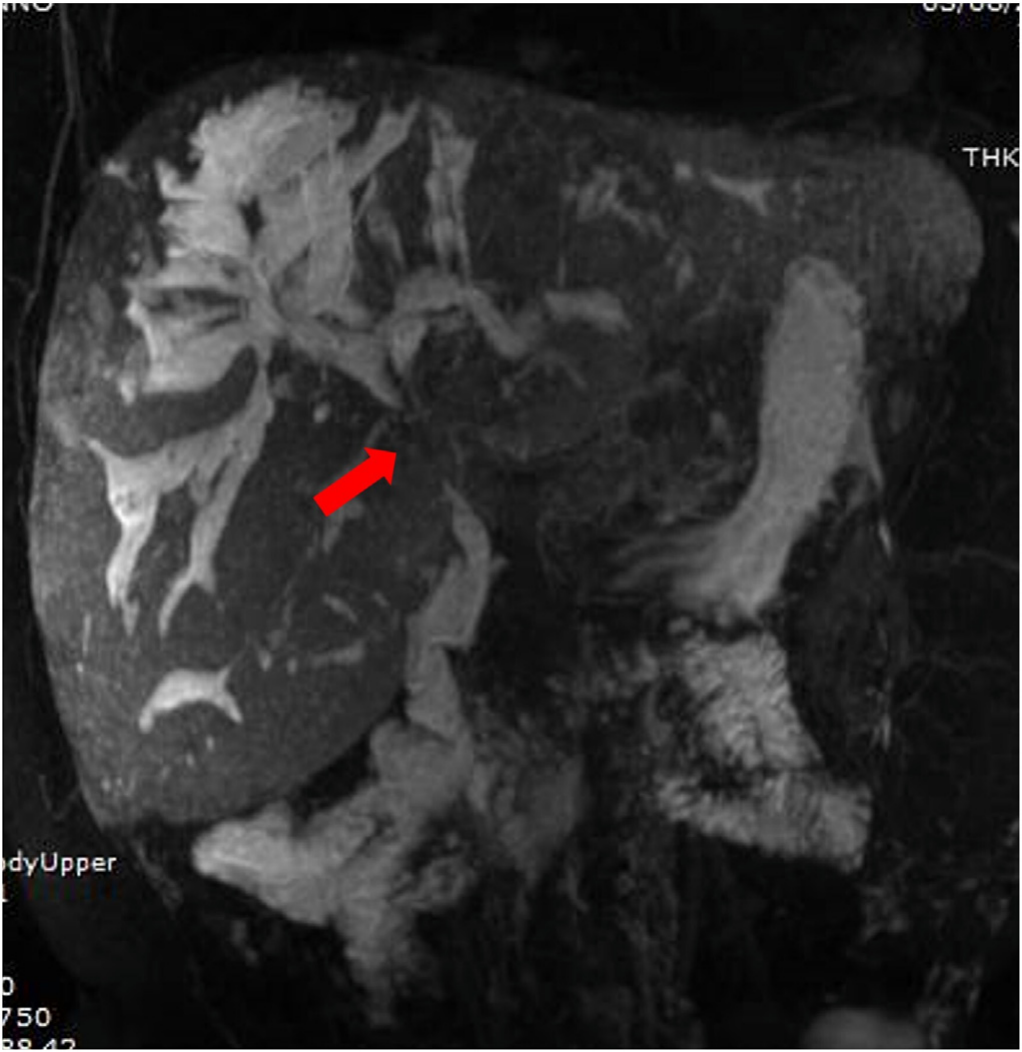
MRCP showing evidence of common bile duct stenosis (*arrow*).

During ERCP, we performed retrograde cholangiography, which confirmed the presence of a narrowing of the common hepatic duct and the proximal common bile duct (Fig. 2). We performed a complete sphincterotomy and subsequently introduced the cholangioscope (Video 1, available online at www.videogie.org).

**Figure 2:**
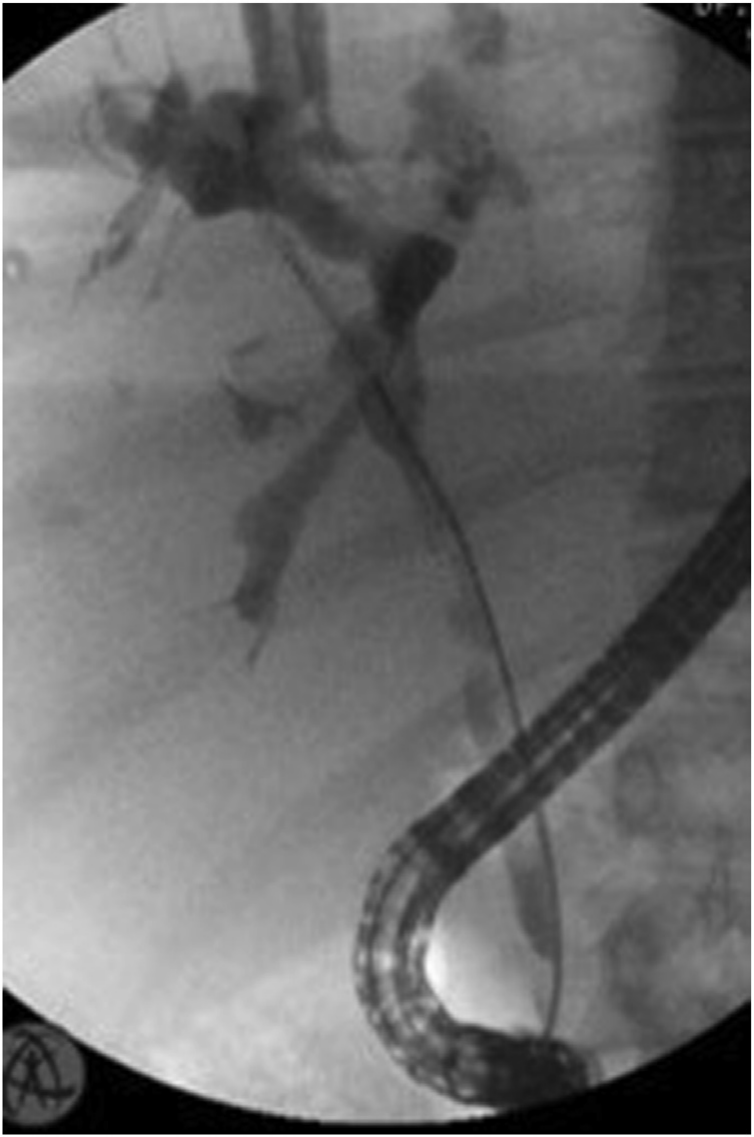
Cholangiography performed during ERCP confirming the presence of common bile duct stenosis.

Once inside the common bile duct, we did not find any stenosis, and the cholangioscope was able to reach the segmental branches of the biliary tree. We observed a common bile duct completely patent but deformed in oval shape, probably due to the surrounding liver fibrosis; we also found fibrous bridges of the intrahepatic ducts, probably secondary to previous cholangitis. Therefore, given the lack of significant stenosis of the biliary pathway and given a good outflow of contrast medium on retrograde cholangiography, we adopted a clinical management of this patient, who was treated with antibiotic therapy with resolution of symptoms and improvement in laboratory parameters.

Usually, cholangioscopy in Caroli disease is reserved to guide lithotripsy in patients with bile duct stones.^3^ Even if cholangioscopy is seen as the most sensitive method to evaluate the nature of uncertain stenosis,^4^ there is still a lack of literature regarding its use in this kind of patient.

The aim of our video is to point out that, despite the high sensitivity and specificity of MRCP for the diagnosis of Caroli disease, its sensitivity in the diagnosis of adverse events such as stenosis may not be as robust. In this case, the use of cholangioscopy led to the correct diagnosis and avoided unnecessary treatments.

## Disclosure

The authors disclosed no financial relationships relevant to this publication.

## Supplementary data

Video 1 Cholangioscopy performed during ERCP.
